# 'Issues of equity are also issues of rights': Lessons from experiences in Southern Africa

**DOI:** 10.1186/1471-2458-7-14

**Published:** 2007-01-26

**Authors:** Leslie London

**Affiliations:** 1 Health and Human Rights Division, School of Public Health and Family Medicine, University of Cape Town Health Sciences Faculty, Anzio Rd, Observatory, 7925, South Africa

## Abstract

**Background:**

Human rights approaches to health have been criticized as antithetical to equity, principally because they are seen to prioritise rights of individuals at the expense of the interests of groups, a core tenet of public health. The objective of this study was to identify how human rights approaches can promote health equity.

**Methods:**

The Network on Equity in Health in Southern Africa undertook an exploration of three regional case studies – antiretroviral access, patient rights charters and civic organization for health. A combination of archival reviews and stakeholder interviews were complemented with a literature review to provide a theoretical framework for the empirical evidence.

**Results:**

Critical success factors for equity are the importance of rights approaches addressing the full spectrum from civil and political, through to socio-economic rights, as well as the need to locate rights in a group context. Human rights approaches succeed in achieving health equity when coupled with community engagement in ways that reinforce community capacity, particularly when strengthening the collective agency of its most vulnerable groups. Additionally, human rights approaches provide opportunities for mobilising resources outside the health sector, and must aim to address the public-private divide at local, national and international levels.

**Conclusion:**

Where it is clear that rights approaches are predicated upon understanding the need to prioritize vulnerable groups and where the way rights are operationalised recognizes the role of agency on the part of those most affected in realising their socio-economic rights, human rights approaches appear to offer powerful tools to support social justice and health equity.

## Background

Despite growing advances in medical technologies, global health status inequalities continue to persist [1-6]. Developing countries are faced with declining expenditures on health and social services, increasing burdens posed by both communicable and non-communicable diseases and economic systems that are not oriented to fostering sustainable development for the poorest and most marginalized [7,8]. In recognition of the social causation of these health trends, the World Health Organisation (WHO) established a Commission on the Social Determinants of Health, reflecting a global concern for the persistence of, and, in some cases, growth in global health inequities [9].

Under these circumstances, does the discourse of human rights offer opportunities for public health practitioners to better negotiate conflicting needs in restructuring health care in countries in transition? Human rights approaches are increasingly cited as important for translating global treaty commitments into health programmes [10,11], in community mobilization to end oppressive conditions harmful to health [12], and in developing appropriate HIV/AIDS intervention programmes [13]. Indeed, some have argued that the attainment of the Millennium Development Goals is not feasible without a commitment to human rights [14].

However, when it comes to the practice of public health, there appears to be a deep-seated ambivalence around whether human rights are really compatible with effective, efficient and equitable health policies [15,16]. This is particularly evident in debates around expanding access to anti-retroviral (ARV) treatment and the potential adverse impact on health equity [17,18] and, more recently, in proposed moves to introduce "routine" testing for HIV in an effort to increase the numbers on ARV treatment [19,20].

Human rights, when framed as entitlements, could be seen to impact negatively on resource allocation by favouring individuals over the welfare of the community, to the detriment of equity [21-23] or contribute to health system inefficiency. For example, South Africa's Minister of Finance, responding to calls for a Treatment Plan for HIV in South Africa, was quoted as arguing that money should rather be spent on poverty relief and building schools than on anti-retroviral drugs, which, in his opinion, were a "waste of very limited resources." [24]

For this reason, rather than automatically assuming that public statements on the links between human rights and health are evidence of consonance, careful analysis should be able to demonstrate why and how this is the case [25]. Otherwise, lofty intent to realize human rights in health will inevitably trip up on the reality of utilitarian public health culture. Despite more than a decade of work on the links between human rights and health [26], only recently have the conceptual links between health equity and human rights begun to receive detailed elaboration to facilitate operationalisation in public health practice [27,28].

This paper reports on the findings of research conducted by the Network for Equity in Health in Southern Africa (EQUINET) to explore the potential synergies between health equity and human rights-based approaches to health [29]; in particular, to identify the specific mechanisms by which human rights can serve to promote health equity.

## Methods

A case-based approach selected three examples to illustrate health rights approaches (Table 1) based upon: a) spread of cases across the region; b) applicability across the region; c) illustrative of different ways in which social mobilization links to human rights approaches; d) accessibility through EQUINET networks of the organizations that were central to each case study.

**Table 1:** Case Studies selected for inclusion

Case	Motivation
1. Treatment Access for HIV: Struggles in Southern Africa (TAC and the Pan African Movement)	The case study illustrates numerous aspects relevant to equity and human rights, as well as providing an example of a successful civil society mobilisation. It raises issues of both legal and advocacy approaches to rights; it touches directly on equity in resource-poor environments; it raises health system concerns; the material is easily available; its lessons may be relatively easy to generalise even if the struggle's successes are not; the relationship between civil society mobilisation and the state/its policy choices will be obvious.
2. Patients' Rights Charters (South Africa, Malawi and Zimbabwe)	Patients' Rights Charters are a commonly used model for promoting the right to health care; it is a consumerist approach to improving quality of health services; it directly addresses health as a socio-economic right; it may or may not be linked to mobilising strategies; it commonly presumes success when it may not have high impact, which itself is a lesson worth exploring – i.e. the limitations of Charters may be as important as any successes; in the implementation a Charter, the role of public participation would be critical.
3. Community Working Group on Health (Zimbabwe)	Example of broad mobilising approach to health; although much of its work does not explicitly speak a language of human rights, it would be useful to tease out whether its approach is actually a rights approach; the role of the CWGH in influencing State Policy, particularly pro-poor choices; leverage over resources outside the health sector, etc. Perhaps comparisons to be made to other developing country examples (e.g. in Brazil)

Data collection took place through a mix of archival research, review of published and unpublished articles and documents (web based and hard copy) and two rounds of interviews with selected key informants. In the first round, members of the organizations involved in each case study were interviewed; in the follow-up, 4 further key informants outside the organizations were interviewed. The following areas were probed in interviews: links between civil and political rights, and socio-economic rights; the organization's engagement with the state and how its work builds community engagement; what kinds of rights strategies have been used to promote health equity; global links in their work; and, intersectoral interventions made possible through the adoption of rights approaches. From the responses, key themes were drawn out so as to develop a clearer conceptual understanding of the relationship between health equity and human rights.

Participants in the informant interviews were given the summaries of discussions for their feedback and invited to join a health rights reference group and participate in a review workshop with civil society participants. Participants gave informed consent prior to interview. Ethics approval for the study was obtained from the Faculty of Health Sciences Research Ethics Committee, University of Cape Town.

Because lack of clear definitions frequently result in confusing use of public health concepts [30] and this imprecision may underlie [25] inappropriate critiques of human rights paradigms [16], the study adopted a priori definitions of key concepts as outlined in Table 2.

**Table 2:** Key Concepts for the interface between Human Rights and Health Equity

A "**Public Health Approach**" is that which addresses the health of whole populations, rather than individuals, using population level analyses to identify and implement strategies for improving well-being of communities, groups or whole populations.
"**Equity**" (vertical equity) refers to policies and programmes that aim to address the prevention of health inequalities – differences in health outcomes that are unnecessary, avoidable and unfair, for example, by allocating greater resources to those in greater need. Vertical equity therefore applies to the process of reaching equal outcomes, of allocating greater resources to ensure reductions in health outcome differentials and, by necessity, implies addressing the power imbalances that underlie inequalities in outcomes and processes[27].
**Human rights **take the form of claims that individuals can legitimately exercise on society to various material or social entitlements deemed essential for dignity and well-being. These claims are based on international governmental consensus incorporated in international law. Unlike principles of medical ethics, once a treaty is ratified by a state, it can be held accountable for its conduct. Human rights are indivisible, including both civil and political, and socio-economic, as well as developmental (environmental/ecological) rights.
**Civil and political rights **include traditional freedoms (e.g. of speech, to vote, of movement, etc). **Socio-economic rights **(e.g. housing, health care, education, etc) are entitlements to services or goods that are social in nature. Supposed distinctions between socio-economic, on the one hand, and civil and political rights are increasingly being recognized as a historically-specific political choice driven by the the Cold War. Currently, global policy formation is therefore increasingly acknowledging the indivisibility of all human rights.
A "**Human Rights Approach**" embraces four elements[31,35]: 1. The use of human rights standards and norms to develop policy and programmes 2. The use of human rights standards and norms to analyse and critique government performance, sometimes combined with a monitoring function 3. The use of human rights standards and norms to facilitate redress for those who suffer violations of their rights. 4. The use of human rights standards and norms to support advocacy and civil society mobilization.
**Health as a human right **is articulated both as access to health care and as the right to health creating-conditions (such as housing, education, a safe environment, etc) in national and international statutes. Government's core obligations to realising the right of access to health care is elaborated in General Comment 14 issued by the United Nations Committee for Economic, Social and Cultural Rights[36].

## Results

The Treatment Access Campaign (TAC) started in 1998 as an advocacy group for people with HIV/AIDS to "campaign for greater access to treatment for all South Africans, by raising public awareness and understanding about issues surrounding the availability, affordability and use of HIV treatments." [33] Initially inspired by similar rights-oriented HIV organizations in the developed world, TAC rapidly developed into a broad-based social movement that has significantly advanced treatment access both in South Africa and in the region, facilitating the establishment of the Pan-African HIV/AIDS Treatment Access Movement. TAC's work has been at the centre of a robust civil society debate in South Africa around the provision of antiretrovirals, in which considerations of effectiveness, equity and efficiency have been prominent [23,24,34-36]. The TAC has also been instrumental in supporting civil society groups in a campaign for a basic social security grant (known as a Basic Income Grant) as a poverty alleviation strategy, and forming alliances to campaign for health system reform.

The Malawian Patients' Rights Charter (PRC) emerged following an advocacy training programme hosted by a foreign NGO in 2000, attended by a range of civil society participants who subsequently established the Malawi Health Equity Network (MHEN). While initial interest was directed at tackling conditions of service for health workers, the network shifted focus to patient advocacy, because of the seeming insurmountability of labour relations difficulties in the health sector. By doing so, it drew in a broader constituency, including professional associations and statutory councils, as well as HIV and consumer advocacy NGOs. The MHEN programme on patient rights focused on the minimum rights available to patients when attending a health service and through iterative interactions with parliamentarians, produced a Charter, which was submitted to government in early 2003. However, because the MHEN relied on leadership coming from the Ministry of Health in bringing the Charter to finality, progress in implementation has all but ceased since the Charter's submission. [29] Organizational difficulties due to ministerial restructuring meant that key meetings could not be held and inclusion of very senior public servants (such as, amongst others, the Permanent Secretary for Health) exacerbated difficulties in coordinating such a high level Task Team.

The Community Working Group on Health (CWGH), a network of membership-based civic and worker organizations in Zimbabwe, was formed in early 1998 in response on to an ongoing decline in the quality of health services, increasing poverty, and industrial action by health workers protesting worsening conditions of service [37,38]. These developments followed the introduction of Economic Structural Adjustment Programmes that eroded post-independence health status gains achieved through heavy investments in pro-poor policies [39]. The CWGH was therefore formed to strengthen civil society capacity to engage with government over health policies through advocacy and networking. It has substantial rural presence (health committees in 21 out of 58 districts in Zimbabwe) and provides a channel for interaction between health care providers and civic organizations, enabling community input to policy processes through advocacy that seeks to reverse or at least halt government's relinquishing of its commitments to equity in health. It has set up opportunities for rural health committees to provide input to Parliamentary structures and participates in oversight of a national AIDS levy established in Zimbabwe to finance various HIV/AIDS activities. The levy was introduced in 1999 and is based on a 3% levy of all taxable income that is routed into a National AIDS Trust Fund, managed by a National AIDS Council.

The case studies illustrate that civil society campaigns for health work most effectively when emphasising the invidivisibility of civil and political, and socio-economic rights (Table 3). For example, the TAC's lobbying for treatment access has also enabled redress of discrimination against people with HIV, through links with legal advocacy groups. Similarly, the CWGH has addressed socio-economic rights under the rubric of service delivery, whilst simultaneously referring members to legal groups involved in defense of civil and political rights. An informant, commenting on the role of the PRC in Malawi, observed:

**Table 3:** Key themes from the case studies: Human rights, health equity and community engagement

• Rights alone are not enough, but need to be coupled with community engagement
• Rights, appropriately applied, can strengthen community engagement
• Rights, conceived in terms of agency, are the strongest guarantors of effective equity-promoting impacts
• Rights should strengthen the collective agency of the most vulnerable groups
• Rights approaches should aim to address the public-private and global divides in relation to Human Rights
• Information and Transparency are key to human rights approaches that build equity
• Human rights approaches provide additional opportunities for mobilising resources outside the health sector

"... when you talk about ... patients' rights, it is something that emerges from several factors ... Like literacy levels, and also geographical ...and material accessibility, and availability of information in rural areas – it's not there. And of course the socio-economic status of the patient determines exposure to different information. So there is a strong linkage between patient rights, socio-economic status, and general human rights."

Evans et al [2] make the link more directly to health equity by pointing out that undemocratic societies characterized by corruption, violence and discrimination are more likely to demonstrate higher inequities in health than those where "respect for human rights, transparency and opportunities for civic engagement" flourish. Health equity therefore requires a conception of rights that operationalises the indivisibility of the full spectrum of human rights.

### Theme 1: Rights alone are not enough, but need to be coupled with community engagement

All three case studies illustrate, either by example or by implication, the importance of broadening rights approaches to embrace active community engagement. One informant described the TAC as "an interesting combination of a rights based movement that also relies on grassroots mobilization. The pressure is through the courts, through the media, as well as in the communities, and on the streets. It is a kind of multipronged approach."

The TAC has used the South African constitution's commitment to socio-economic rights to force the state to provide antiretrovirals (ARVs) for the Prevention of Mother-to-Child Transmission of HIV [23,34,35]. However, while legal strategies have been one pillar of the TAC's successes, TAC has consistently matched legal strategies with grassroots mobilisation in ways that are mutually reinforcing, arguing that "human rights arguments and legal action alone are of limited use. It is crucial to combine them with mass mobilization, including human rights awareness campaigns." [34] TAC has explicitly invested organizational effort in workshops to train members in understanding health rights and treatment access as a right, as consistently evident in TAC campaign media.

By comparison, a seminal case highlighting the justiciability of socio-economic rights in South Africa, the Grootboom case, which involved the halting of evictions of a community living in an informal settlement outside Cape Town in 2000, was hailed for its important legal precedent [40]. However, the court decision produced no grassroots impacts because there was no community action to complement the legal challenge. Thus, despite the court decision, no major shifts in housing policy eventuated, nor have communities and groups in most need been able to make use of the decision to improve their situation [41]. Illustrative of TAC's arguments, therefore, legal strategies alone are of limited impact without popular mobilisation.

### Theme 2: Rights, appropriately applied, can strengthen community engagement

The idea that a charter of patients' rights could assist in realizing better quality health care underlies much health planning [42,43]. However, it is less obvious how such a charter would operationalise users' rights. What emerged from the case studies was that the charter's most valuable role would be to provide community members with a standard for negotiating quality of care with providers at their facilities in the context of meaningful community participation.

"I think it will be even more important, within this new sector wide approach, to have such a charter. So that community members know what is their right, and how they can negotiate that with the health workers, or the district head office, or the district health management teams."

However, in the way the PRC was developed in Malawi, as a technical process without community input, it did not build organization around health. Indeed, evidence suggests that, once submitted to government, the PRC was allowed to fade from a development agenda.

"... in terms of the process, somehow, there was a loose link between the community members, and the people who were facilitating it. Plus, also, there wasn't the follow up, or linkage, between the facilitators, and the Minister of Health officials, who, according to my knowledge, took it up and said, okay, we need to institutionalize it, and then from there, the momentum started decreasing slowly, and now there is silence about it..."

Opportunities to challenge this demobilisation through community participation structures in Malawi were reported as severely restricted by the legacy of the previous Banda government, when civic structures were used to exercise political patronage rather than play active roles on behalf of civil society. Health Committees were therefore distrusted as vehicles for community voice. In contrast, the CWGH's work illustrates effective mobilisation around entitlements to health services using community health committees to enhance civil society capacity to input to local facility management and national policy. For example, the CGWH has brought community preferences into decisions regarding the distribution of the national HIV levy and facilitated community inputs to the Parliamentary Portfolio Committee on Health [63]. Even under difficult political conditions prevalent in Zimbabwe over the past decade, engagement with health rights, albeit in the discourse of service delivery, opened spaces for civil society to advance the needs of the most vulnerable communities, while at the same time, building community organization. Similarly, the use of rights approaches has both advanced the TAC's treatment access objectives whilst simultaneously helping to recruit members, strengthen the organization and build alliances outside of the health sector.

Thus, in pursuing health equity, human rights, appropriately applied, can strengthen community engagement to achieve health equity.

### Theme 3: Rights, conceived in terms of agency, are the strongest guarantors of effective equity-promoting impacts

Diderichsen et al, identify four levels at which powerlessness lies at the root of health inequalities: social stratification; differential exposure based on social stratification; differential vulnerability given an exposure; and differential consequences [44]. Attempts to redress inequities therefore have to engage with questions of power [28,30] and it is not surprising that the public health community is increasingly returning to approaches that revive the notion of community agency in public health practice. Rather than framing the poor as candidates for protection or redistributive policies by a benevolent state, commentators have called for a "new" public health that takes seriously its commitment to community empowerment [45-49]. This agency is illustrated in all three case studies, where mechanisms were present to facilitate active community interaction with policy makers. Interactions were either collaborative (e.g. committees to develop a charter or a resource distribution decision on a national levy) or combative (e.g. a courtroom challenge for treatment access for HIV) but were all essential to achieving equitable outcomes. For example, one informant described the impact of the work of TAC as follows:

"TAC does draw a link between people's health and to the degree to which they network and mobilize, and the degree in which they are involved in other community processes. Just by nature of the fact that they are increasing awareness and activity around people's health, and in this instance, specifically around HIV/AIDS, it is drawing a link between a kind of evident ability to impact on your world and a sense of self advocacy."

Working towards health equity, therefore, requires rights-based approaches that provide opportunities to all people (not just the most vocal) to input to policy and its implementation so as to reverse the social exclusion that is the key pathway between social inequality and health inequities [50]. More recently, attention has focused on the role of active citizenship as the key element for translating hard-won rights to ARV access into reality [36]. Of course, the strength of rights-based approach is that is can simultaneously foster community agency whilst still holding government to account for its human rights obligations, thereby avoiding the abrogation of state responsibility for the welfare of all its citizens, so typical of neoliberal constructions of the modern state.

### Theme 4: Rights should strengthen the collective agency of the most vulnerable groups

In describing the work of the TAC, informants emphasized the importance of collective actions. For example:

"These are highly politicized activists whose strength is in the organization, in the fact that individuals are organized as a group, and as a movement."

The fact that agency is strengthened not for individuals but as part of a vulnerable group is critical to challenging the powerlessness [50] underlying health inequalities. For example, the advocacy work of the TAC and CWGH in bringing community preferences to bear on national health policy, has reversed the "thinness of reserves" [44] characteristic of groups suffering health inequities. Analogous to role of human rights in enabling individuals to realize their capabilities [51], is the role of community mobilization using rights strategies to provide citizens with collective avenues to ensure access to the resources needed for health. In the words of one informant:

"This is not just self, but a collective community type advocacy and health. So there is this incredible communication around what we can achieve if we are mobilized and we can network, stay focused and work together, and, in particular, the direct benefits of a certain campaign around health. There are all these kinds of side benefits that also come with it. People do realize the impact that can be made when we work together ..."

Moreover, rights approaches that prioritize the most vulnerable and provide people with opportunities for agency, intrinsically address an equity promoting agenda by privileging the experiences of poor and marginalized groups [52]. Such views, however, are not uniform. For example, Muller argued that "The fact that TAC has the financial clout to take the government to court does not mean that its case is more important than that of people living in rural poverty." [22] Implicit in this view is the notion that the TAC is a kind of aristocracy amongst marginalized people. However, this view represents an ahistorical interpretation of rights [53] that ignores the fact that rights have emerged not just from legal strategies but from a combination of political pressure, grassroots mobilisation and activism [54]. As Valente [55] (1998) argues, "... the history of human rights ... has been tortuously and painfully built from conflict to conflict, at the cost of the suffering, pain, struggle and lives of the great majority of anonymous human beings ..." (p180). A human right approach must engage the dimension of power [56], since social justice and anti-discrimination are key dimensions of its framework. Out of this challenge to power, emerges a synchrony with health equity frameworks that seek to redress health differences that are unnecessary, avoidable and unfair [57].

### Theme 5: Rights should aim to address public-private and global divides in relation to Human Rights

In the context of the undermining of national sovereignty by globalisation, rights approaches have afforded opportunities for global solidarity and action to strengthen pro-poor policies at national and international levels [23,35]. For example, the TAC's support of the South African government in defending its pharmaceutical legislation from legal attack by industry drew on unprecedented global solidarity mobilized through the TAC's international networks and played an important role in defeating the industry's opposition to the legislation, forcing industry to reduce drug prices for antiretrovirals [58]. South Africa's experience in rights campaigns for treatment access has also played a key role in building a regional treatment access movement in Southern Africa, where needs are as desperate but resources far more limited than in South Africa [59].

Similarly, at international level [58], collaboration during trade negotiations between NGO's aligned to treatment access initiatives and southern states was able to ensure that access to essential medicines was addressed at the Doha round of WTO talks. In this way, community mobilisation has been able to reinforce, and be consolidated by, action at the level of state power, successfully harnessing potential synergies between formal and constituent power, even in an environment of market-driven disempowerment of nation states [35]. Rights approaches have therefore increased opportunities for mobilising support through the global human right movement, which has, in turn, strengthened state capacity to regulate in favour of pro-poor policies.

Besides private sector industry, rights approaches also place the spotlight on the behaviour of donor agencies [60,61] and non-state actors [60]. For example, one respondent drew attention to the influence of donors on policy development:

"Yes ... there are a lot of linkages ... at national level, how different factors, political, social, might influence the work to go ahead or not, and also globally, there are also several factors, it's an issue of power. Maybe the donors, they have a certain preference, they might not take it as an important issue."

A construction of rights as being simply about what entitlements citizens can expect from government is therefore neither helpful for equity, nor grounded in the political realities of globalization. Indeed, multinational companies are increasingly being expected to uphold rights, such as the right to participation by employees and communities affected by their operations [61,62]. In its work on pharmaceutical access, the TAC has shown how it is possible to expand the purview of rights to address public-private inequalities that drive health inequities. Moreover, the inclusion of private providers' obligation to provide emergency care in the Malawian PRC reflects how rights approaches, even in less high-profile settings, can begin to tackle public-private inequalities using community agency and advocacy language. However, such strategies to extend the envelope of what rights approaches can do, will only succeed in the context of strong civil society action.

### Theme 6: Information and Transparency are key elements for health equity

As both a right in itself and an enabling mechanism to realize other rights, access to information plays a key role. On the individual level, information is key to countering powerlessness:

"... when you talk about the Patients' Charter, patients' rights, it is something that emerges from several factors. Because sometimes, why patients might feel powerless, is because of lack of information."

But it is also at a collective level, that information empowers civil society to drive the shifts in political will required for policy change [55]. Systems that maximize transparency and accountability offer the most likely opportunities for community engagement and meaningful input. For example, the TAC have mobilised their own 'experts' to develop positions on key HIV-related debates, so that information is available to grassroots membership through its media and workshops, and disseminated through campaigns to the public. The CWGH have enlisted researchers to access information on health conditions and services to support campaigs for health equity in Zimbabwe. Use of research has occurred dialectically, strengthening civil society's ability to engage with the state and the private sector in the pursuit of health equity goals.

Conversely, absence of information and transparency undermines community agency, and drives conflict and distrust that undermines redress of inequity. For example, the closure of channels of access to information regarding Poverty Reduction Strategy Papers in Malawi has been interpreted as reversing gains made through interaction with policy-makers over the PRC [29].

Rights to information are therefore key to operationalising the right to health.

### Theme 7: Human rights approaches provide additional opportunities for mobilising resources outside the health sector

Human rights approaches also facilitate mobilization across sectors. A legal victory in South Africa's courts relating to the right to housing was key to bolstering the TAC's rights-based arguments for ARV access, and the CWGH has been able to integrate housing and sanitation issues easily in its health advocacy. The TAC's central role in contributing to a broad-based coalition advocating a Basic Income Grant as a social security measure illustrates not only a grasp of the multisectoral origins of health but also a strategic capacity to develop alliances across a range of sectors, including the Trade Union movement, churches and other elements of civil society. The breadth of these alliances (i.e. with the 'non-vulnerable' in organized labour, academia, research, parliamentarians and health care providers) has been extremely effective through both intellectual (research data) and advocacy (media, protest mobilization), countering the social stratification implicit in the vulnerability underlying health inequities [44]. As argued by one informant:

"... government ... has been concerned with attracting international capital, it has been concerned with the so-called first economy, but not the 'second economy', the informal sector, the hopeless, the jobless, the people who work on the side of the road, waiting for job opportunities. So there is economic injustice ... I think the TAC forms part of a broader agenda to address that."

## Conclusion

**Figure 1:**
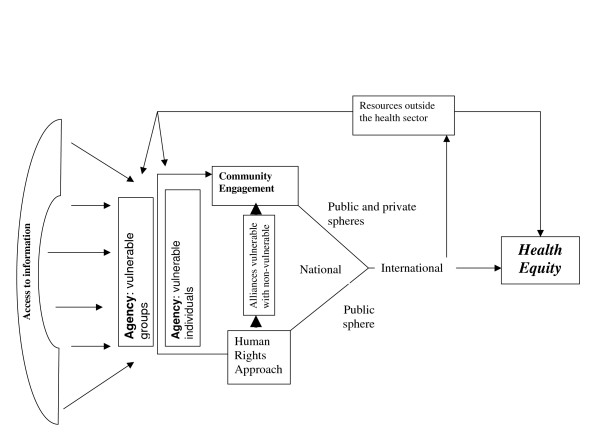
Human Rights approaches, Agency and Health Equity: A Model.

Where it is clear that rights approaches are predicated upon understanding the need to prioritize vulnerable groups, where the way rights are operationalised recognizes the role of agency by those most affected, and where rights are conceived as the complete spectrum of civil and political, through to socio-economic rights, human rights approaches appear to offer powerful tools to support social justice and health equity (figure 1). Public health concerns for equity then become entirely consonant with human rights-based strategies and tactics. The synergy between public health and human rights in relation to equity lie less in the pursuit of individual rights but rather in the way social processes and consciousness are given the opportunity to the interface with the state in ways that that secure collective rights.

## Competing interests

The author(s) declare that they have no competing interests.

## Authors' contributions

The author conceived the research idea, oversaw data collection and undertook analysis and write up of the material, including revisions recommended by reviewers.

## Pre-publication history

The pre-publication history for this paper can be accessed here:


